# Genomic characterization of carbapenem and colistin-resistant *Klebsiella pneumoniae* isolates from humans and dogs

**DOI:** 10.3389/fvets.2024.1386496

**Published:** 2024-05-21

**Authors:** Ruttana Pachanon, Nwai Oo Khine, Nathita Phumthanakorn, Thidathip Wongsurawat, Waree Niyomtham, Tanittha Chatsuwan, David J. Hampson, Nuvee Prapasarakul

**Affiliations:** ^1^Department of Veterinary Microbiology, Faculty of Veterinary Science, Chulalongkorn University, Bangkok, Thailand; ^2^Department of Pre-Clinic and Applied Animal Science, Faculty of Veterinary Science, Mahidol University, Nakhon Pathom, Thailand; ^3^Siriraj Long-Read Lab (Si-LoL), Faculty of Medicine Siriraj Hospital, Mahidol University, Bangkok, Thailand; ^4^Department of Microbiology, Faculty of Medicine, Chulalongkorn University, Bangkok, Thailand; ^5^School of Veterinary Medicine, Murdoch University, Perth, WA, Australia; ^6^Center of Excellence in Diagnosis and Monitoring of Animal Pathogens (DMAP), Bangkok, Thailand

**Keywords:** *Klebsiella pneumoniae*, carbapenem resistance, colistin resistance, plasmid-mediated resistance, whole genome analysis

## Abstract

**Introduction:**

Carbapenem and colistin-resistant *Enterobacteriaceae*, including *Klebsiella pneumoniae*, have become a growing global concern, posing a significant threat to public health. Currently, there is limited information about the genetic background of carbapenem and colistin-resistant *K. pneumoniae* isolates infecting humans and dogs in Thailand. This study aimed to characterize carbapenem and colistin-resistant genes in six resistant *K. pneumoniae* clinical isolates (three from humans and three from dogs) which differed in their pulse field gel electrophoresis profiles.

**Methods:**

Matrix-assisted laser desorption ionization-time of flight mass spectrometry (MALDI-TOF MS), antimicrobial susceptibility testing, and whole-genome sequencing were employed to identify and analyze the isolates.

**Results and discussion:**

All six isolates were carbapenemase-producing *K. pneumoniae* isolates with chromosomally carried *bla*_SHV_*, fosA, oqxA* and *oqxB* genes, as well as nine to 21 virulence genes. The isolates belonged to five multilocus sequence types (STs): one isolate from a human and one from a dog belonged to ST16, with the other two human isolates being from ST340 and ST1269 and the other two dog isolates were ST147 and ST15. One human isolate and two dog isolates harbored the same *bla*_OXA-232_ gene on the ColKP3 plasmid, and one dog isolate carried the *bla*_OXA-48_ gene on the IncFII plasmid. Notably, one human isolate exhibited resistance to colistin mediated by the *mcr-3.5* gene carried on the IncFII plasmid, which co-existed with resistance determinants to other antibiotics, including aminoglycosides and quinolones. In conclusion, this study provides a comprehensive characterization of both chromosome- and plasmid-mediated carbapenem and colistin resistance in a set of *K. pneumoniae* clinical isolates from unrelated humans and dogs in Thailand. The similarities and differences found contribute to our understanding of the potential widescale dissemination of these important resistance genes among clinical isolates from humans and animals, which in turn may contribute to outbreaks of emerging resistant clones in hospital settings.

## Background

*Klebsiella pneumoniae* is designated as a critical pathogen of priority by the World Health Organization (WHO) (1), and it poses a significant threat to public health. This opportunistic bacterium commonly infects immunocompromised individuals in hospital settings, leading to various severe infections such as pneumonia, sepsis, urinary tract infections (UTIs), bacteremia, meningitis, and pyogenic liver abscesses (2). The emergence of resistance to antibiotics, particularly colistin and carbapenem, further complicates the treatment of *K. pneumoniae* infections in both human and animal populations.

The prevalence of carbapenem resistance in *K. pneumoniae* can vary depending on the geographical region and the specific population being studied. Generally, carbapenem-resistant *K. pneumoniae* (CRKP) strains have become a significant concern for both human and animal health (3–5). The prevalence of CRKP strains can be high in human healthcare setting in some regions, leading to increased morbidity and mortality rates among infected individuals (3). In animal settings, including in livestock and companion animals, CRKP isolates also have been reported, although the prevalence tends to be lower compared to in human populations (6, 7). Nevertheless, the presence of CRKP in animals raises concerns about potential transmission to humans through direct contact, environmental contamination, or foodborne routes (5). The One Health approach recognizes the interconnections between human, animal, and environmental health and emphasizes the importance of surveillance, prevention, and control measures to mitigate the spread of carbapenem resistance between different reservoirs (6).

Phenotypic methods are typically the first step in detecting carbapenem resistance. These methods involve performing antimicrobial susceptibility testing (AST) using disk diffusion or broth microdilution methods. Carbapenem-resistant strains exhibit reduced susceptibility or resistance to carbapenem antibiotics, such as imipenem, meropenem, or ertapenem (8). Carbapenemase enzymes are the most common mechanism of carbapenem resistance in *K. pneumoniae*. Several methods can detect carbapenemase production, including the Modified Hodge Test (MHT), Carba NP test, and Carba-R assay (9, 10). These tests detect the presence of carbapenemase enzymes by assessing the ability of the organism to hydrolyze carbapenem antibiotics. CRKP strains often possess various carbapenemase encoding genes, such as NDM-1, OXA variants, and KPC, which contribute to their resistant phenotype (11, 12).

Carbapenem resistance in *K. pneumoniae* is predominantly associated with the presence of carbapenemase genes carried on large, self-conjugative plasmids, although the *bla*_OXA-232_ gene (13) encoding resistance to carbapenems is commonly found on small ColE-like plasmids. Furthermore, the co-existence of multiple carbapenemase genes within a single isolate, such as *bla*_NDM*-*1_ and *bla*_OXA-232_ in sequence type (ST) 14, has been reported (14). Isolates of the globally prevalent *K. pneumoniae* ST16 also demonstrate diverse antimicrobial resistance profiles. For example, an outbreak of nosocomial infections caused by ST16 *K. pneumoniae* carrying *bla*_CTX-M-15_ occurred in Sweden and Denmark (4), while the co-occurrence of *bla*_NDM*-*1_ and *bla*_OXA-232_ in ST16 *K. pneumoniae* was identified in Italy (15). In a study in Thailand, the two STs that were most commonly found in *K. pneumoniae* from clinical affected humans were ST16 (*n* = 15) and ST231 (*n* = 14) (16).

The current study aimed to enhance understanding of the occurrence of antimicrobial resistance genes in *K. pneumoniae* isolates from clinically affected humans and dogs in hospital settings in Thailand. Specifically, we sought to elucidate the genetic mechanisms underlying carbapenem resistance in *K. pneumoniae*, assess the co-occurrence of other antimicrobial resistance genes, and compare the genetic characteristics of isolates from humans and dogs to help determine the possibility of transmission. Resistance to colistin was of interest as studies have revealed the presence of the *mcr-3.5* gene in the IncFII plasmid type of pig-derived *Escherichia coli* isolates in Thailand and other countries (17–19). To date, the prevalence of *K. pneumoniae* in clinically affected companion animals in Thailand has not been elucidated. In this work, we outline the prevalence, characteristics, molecular typing, and whole genome sequence data for antibiotic resistant *K. pneumoniae* isolates from clinically affected companion dogs and humans in Hospital settings.

## Materials and methods

### Sample collection and identification

In 2020, 10 CRKP isolates from humans that met our inclusion requirements, which included being clinically resistant to the carbapenem drugs tested (imipenem, meropenem or ertapenem) were selected for study. They had been cultured from the urine of human patients with urinary tract infections (UTIs) at the King Chulalongkorn Memorial Hospital, Chulalongkorn University, Bangkok as part of routine diagnostic investigations. Another three isolates were collected from dogs with UTIs at the Small Animal Teaching Hospital, Faculty of Veterinary Science, Chulalongkorn University. All the isolates were obtained during the same month in 2020. The humans and dogs were all from different households. Isolates picked from MacConkey agar were identified as *K. pneumoniae* using IMViC biochemical tests and MALDI-TOF MS (Bruker-Daltonics, Bremen, Germany) (20). The isolates then were tested for antimicrobial susceptibility using the Vitek 2^®^ (bioMérieux, France) and were subjected to *Xba1*-PFGE macrorestriction analysis using a previously described protocol (21). Briefly, the genomic DNA from the 13 CRKP isolates was digested with the restriction enzyme *XbaI* (Thermo Scientific). The Bio-Rad CHEF-DRIII system was used for gel electrophoresis, set with a 200 V field at an angle of 120° and run for 17–20 h: *Salmonella* serovar Braenderup H9812 DNA was incorporated as a standard control. Dendrograms were visualized by using the GeneTool program (Syngene, India) and analyzed with the GeneDirectory program (Syngene, India). Isolates showing different PFGE profiles then were subjected to multilocus sequence typing (MLST) by simplex PCR (Supplementary Table S1). Seven housekeeping genes including the beta-subunit of RNA polymerase B (*rpoB*), glyceraldehyde 3-phosphate dehydrogenase (*gapA*), malate dehydrogenase (*mdh*), phosphoglucose isomerase (*pgi*), phosphoporine E (*phoE*), translation initiation factor 2 (*infB*), and periplasmic energy transducer (*tonB*) were amplified (22). The MLST database at http://pubmlst.org/kpneumoniae was used to determine allele and sequence types (STs).

Three *K. pneumoniae* isolates from humans and three from dogs that were all resistant to carbapenem and appeared genetically distinct from each other were selected for whole genomic sequencing and additional analysis. The human isolates were designated KPH1, KPH3, and KPH4 and the dog isolates were KPA1, KPA2, and KPA3. There were no known connections between the six patients. The bacteria were preserved at −80°C in Tryptic Soy broth (Difco) containing 25% glycerol and were recovered on Tryptic Soy agar (Difco) containing 5% sheep blood.

### Antimicrobial susceptibility testing

The six representative *K. pneumoniae* isolates were further tested for their carbapenem susceptibility using the Sensititre Complete Automated AST System (Thermo Scientific, United Kingdom). Following the Clinical and Laboratory Standards Institute (CLSI) standards VET01S (2023) (23), the inclusion criterion was non-susceptibility, which was characterized as resistance or intermediate phenotypes to at least one of the three carbapenems, imipenem, ertapenem, and meropenem. The CRKP isolates also were examined for their susceptibility to colistin, cefoxitin, cefepime, cefotaxime, ceftazidime, cefotaxime/clavulanic acid, ceftazidime/clavulanic acid, temocillin, gentamicin, ciprofloxacin, nalidixic acid, sulfamethoxazole, trimethoprim, tetracycline, tigecycline, chloramphenicol, azithromycin, and ampicillin. Isolates that were resistant to two or more antimicrobials were considered to be multi-drug resistant. The minimum inhibitory concentration (MIC) for colistin was determined by using the broth microdilution technique, with an MIC value of ≥4 μg/mL considered to indicate colistin resistance (23). Antimicrobial susceptibility testing for *mcr* positive *K. pneumoniae* isolates was performed by using the AST-GN 38 test kit in a Vitek2 apparatus (bioMérieux, France) (24). *E. coli* ATCC 25922 was used as the quality control strain for AST.

### DNA preparation and whole genome sequencing

The six *K. pneumoniae* isolates were sub-cultured on Tryptic Soy agar at 37°C for 18 h. Genomic DNA was extracted from the isolates using the ZymoBIOMICS DNA Miniprep Kit following the manufacturer’s instructions, and a Qubit Fluorometer was used to assess the quantity of the extracted DNA. The genomic sequences of the isolates were obtained using the Illumina NovaSeq PE150 platform and MinION (Oxford Nanopore Technologies for long read sequencing).

### Whole-genome analysis and bioinformatics

Paired-end reads were quality filtered to remove adapters and low-quality sequences with quality scores <30 using Trimmomatic v.0.36.5 (25). The associated bioinformatic studies were conducted on the European Galaxy server.^1^ Clean raw reads were assembled and analyzed with Unicycler hybrid assembly (Galaxy Version 0.4.8.0) using the default settings (26). Sequences were analyzed for species identification (KmerFinder 2.1), Multilocus Sequence Type (MLST 1.6), virulence factors (VirulenceFinder 1.2), antimicrobial resistance (ResFinder 2.1), plasmids (PlasmidFinder 1.2) and mobile elements (mobile element finders v1.0.3), using the Center for Genomic Epidemiology (CGE) pipeline (27). Using ABRicate on the Galaxy server, the presence of acquired antimicrobial resistance genes (ARGs) and *K. pneumoniae* virulence factors also were investigated (Galaxy Version 1.0.1). The CARD Resistance Gene Identifier (28) and ARG-ANNOT (Antibiotic Resistance Gene-ANNOTation) (29) databases were employed in this platform, while the VFDB databases were used for virulence genes (30). The NCBI Prokaryotic Genomes Annotation Pipeline (PGAP) and Prokka (Prokaryotic genome annotation) (Galaxy Version 1.14.6) were used to annotate the genomes (31). Screening for biocide resistance genes using the antibacterial biocide and metal resistance gene databases ResDB and BacMet, was undertaken on the BacAnt server (32, 33).

Plasmid sequences were obtained using plasmid finder and annotated by Prokka (Galaxy Version 1.14.6) (31). PlasmidFinder and ResFinder, tools from the Center for Genomic Epidemiology (CGE),^2^ were used for predicting plasmids and genes associated with antimicrobial resistance, respectively. All the reference plasmid and other sequences used in the study were recovered from the NCBI database. VFDB, a tool on the Galaxy Europe server, was used for interpreting virulence genes (34). The pan-genome and single nucleotide polymorphisms (SNPs) were determined using Roary v. 3.13.0 (Galaxy Europe server) (35). For phylogenetic tree analysis, *bla*_OXA-232_ (in isolates KPH4, KPA1, and KPA2) was selected for multiple alignment of nucleotide sequences using MEGA11 software and the maximum likelihood method (100 bootstrap replicates). In addition, the whole genome sequences of the isolates carrying *bla*_OXA-232_ on the COlKP3 plasmid were compared to those of reference strains carrying *bla*_OXA-232_ obtained from Thailand (LC613148.1), Nepal (LC507653.1), Bangladesh (CP096175.1), China (CP097391.1), India (CP079128.1), the Netherlands (CP068856.1), Italy (MH523449.1), the USA (CP006802.1), South Korea (CP031737.1), Germany (CP091603.1), and the United Arab Emirates (MF774791.1).

### Phylogenetic SNP tree analysis

Thirty-four complete genomes of KPC deposited in Genbank at NCBI were selected for comparative analysis. The core genes were analyzed in Roary version 3.13.0, then the find SNP site version 2.5.1 in the Galaxy website (see text footnote 1) was used to align single nucleotide polymorphism (SNP) sequences (36). A phylogenetic SNP tree was constructed in IQTree version 2.1.2 under maximum likelihood and visualized at the iTOL website^3^ (37). SNP distance matrix version 0.8.2 was used to compute distance in SNPs.

### Plasmid conjugation assay

The six *K. pneumoniae* isolates (Donor strains) were examined for conjugative plasmid activity as previously described (38). Transfer frequencies were determined by dividing the number of transconjugants by the number of donor colonies (log of transconjugants on selective media/log of the donor). Colonies of the recipient *E. coli* J53 (Transconjugants) were selected on LB agar (Oxoid, England, United Kingdom) plates containing 200 μg/mL sodium azide and 1 μg/mL imipenem, while *E. coli* ATCC 25922 was used as a control strain. The transconjugant colonies were identified utilizing PCR. Bacterial DNA was extracted using the Thermo Scientific GeneJET Genomic DNA Purification Kit (Thermo Fisher Scientific). A multiplex-PCR for 11 acquired carbapenemase genes (*bla*_IMP_, *bla*_VIM_, *bla*_SPM_, *bla*_GIM_, *bla*_SIM_, *bla*_KPC_, *bla*_NDM_, *bla*_AIM_, *bla*_DIM_, *bla*_BIC_, and *bla*_OXA-48_) was undertaken on the extracted DNA following a previously published protocol (11).

### Data availability statement

The whole-genome sequences of the *K. pneumoniae* isolates were deposited in the GenBank database with the following accession numbers: KPH1 (CP102546-48), KPH3 (CP102552-53 and CP102555), KPH4 (JAOAOE010000001, JAOAOE010000005, JAOAOE010000006, and JAOAOE010000009), KPA1 (CP101877-78, CP101880-81), KPA2 (CP102987-991), and KPA3 (CP102993-995).

## Results

### Antimicrobial susceptibility testing

Two human isolates (KPH1 and KPH3) and two dog isolates (KPA1 and KPA3) were resistant to all the antimicrobial drugs that were tested (Table 1; Supplementary Table S1). All six isolates were resistant to carbapenem (ertapenem), and four (KPH1, KPH3, KPA1, and KPA3) were resistant to imipenem and meropenem. Only KPH4 from a human showed co-resistance to colistin and ertapenem.

**Table 1:** Antimicrobial susceptibility test results for the six *Klebsiella pneumoniae* isolates.

Isolates	Source	β-Lactams										Amino glycosides	Quinolones		Folate pathway antagonists		Tetracyclines		Phenicols	Lipopeptide	Macrolide	Penicillin
		FOX	FEP	FOT	TAZ	F/C	T/C	TRM	ETP	IMI	MERO	GEN	CIP	NAL	SMX	TMP	TET	TGC	CHL	COL	AZI	AMP
KPH1	Human																					
KPH3	Human																					
KPH4	Human																					
KPA1	Dog																					
KPA2	Dog																					
KPA3	Dog																					

Resistant is red, Intermediate is green, and Susceptible is yellow. FOX, Cefoxitin; FEP, Cefepime; FOT, Cefotaxime; TAZ, Ceftazidime, F/C, Cefotaxime/Clavulanic acid; T/C, Ceftazidime/Clavulanic acid; TRM, Temocillin; ETP, Ertapenem; IMI, Imipenem; MERO, Meropenem; GEN, Gentamicin; CIP, Ciprofloxacin; NAL, Nalidixic acid; SMX, Sulfamethoxazole; TMP, Trimethoprim; TET, Tetracycline; TGC, Tigecycline; CHL, Chloramphenicol; COL, Colistin; AZI, Azithromycin; AMP, Ampicillin.

### Genomic characterization of carbapenem resistant *K. pneumoniae*

The six CRKP isolates submitted for whole genome sequencing had a MIC of ≥2 μg/mL for ertapenem or a MIC of ≥4 μg/mL for imipenem or meropenem; they belonged to five different sequence types in MLST and all had different PFGE profiles. Their genome sizes ranged from 5 ~ 5.3 Mb. Information about the CRKP, including their STs, carbapenem-resistant *Enterobacteriaceae* (CRE) genes, and plasmid replicon type is presented in Table 2. All isolates were confirmed as CRKP by possessing NDM-1 (New Delhi metallo- β-lactamase) or OXA (oxacillinase) genes. KPH3 and KPH4 had both types of CRE genes, but with different OXA types. InFIA and IncFIB were common replicon types. A small (6,140 bp) ColKP3 plasmid was found in human isolate KPH4 and dog isolates KPA2.

**Table 2:** Profiles of the six dog and human carbapenem resistant *Klebsiella pneumoniae* isolates detected by PCR.

No.	Bacterial isolate	Source	Sequence type	Carbapenemase genes		Plasmid replicon types
				NDM variants	OXA variants	
1	KPA1	Dog Dog Dog	ST16		OXA-48 OXA-232	IncFIA and IncFIB
2	KPA2		ST147		OXA-232	IncFIB and InC, ColKP3
3	KPA3		ST15	NDM-1		IncFIB and IncHI
4	KPH1	Human Human Human	ST16	NDM-1	OXA-9	IncFIA and IncFIB
5	KPH3		ST340	NDM-1	OXA-1	IncFIA and IncN
6	KPH 4		ST1269		OXA-232	IncFII, IncN, and ColKP3

All six isolates had a similar CG content percentage (57–57.2%), number of CDSs (5,247–5,458), number of rRNAs (24–25), and number of tRNAs (87–91). No CRE or CRISPR genes were present on the chromosome maps. The *bla*_SHV_ family genes encoding beta-lactam resistance were present in all isolates, together with the *fosA5* or *fosA6* fosfomycin-resistance encoding genes. The *oqxA* and *oqxB* genes, encoding an RND-family multidrug efflux pump OqxAB that mediates quinolone resistance, also were identified on their chromosomes.

Between nine and 21 virulence genes were identified in the six CRKP isolates (Table 3). All six shared nine of these genes: *ompA, entA, entB, fepC, yagV/ecpE, yagW/ecpD, yagX/ecpC, yagY/ecpB*, and *yagZ/ecpA*. The virulence type and pattern of the 21-gene cluster contained by human isolate KPH1 and dog isolate KPA1 were identical. These isolates were both members of ST16 and had a similar resistance profile. The *fepG* gene was only found in dog isolate KPA2 (Table 3).

**Table 3:** Comparison of antimicrobial resistance genes and virulence genes on the chromosome of *K. pneumoniae* of human and dog origin.

	Chromosome of human isolates			Chromosome of dog isolates		
	KPH1 (5,321,148 bp)	KPH3 (5,280,364 bp)	KPH4 (3,059,820 bp)	KPA1 (5,270,951 bp)	KPA2 (5,293,381 bp)	KPA3 (5,272,495 bp)
Accession no. (*NCBI)	CP102546	CP102552	JAOAOE010000001	CP101877	CP102987	CP102993
Resistance genes						
β-lactam	*bla*_SHV-26_ *bla*_SHV-78_ *bla*_SHV-98_ *bla*_SHV-145_ *bla*_SHV-179_ *bla*_SHV-194_ *bla*_SHV-199_ *bla*_CTX-M-15_	*bla*_SHV-182_	*bla*_SHV-11_ *bla*_SHV-13_ *bla*_SHV-70_	*bla*_SHV-26_ *bla*_SHV-78_ *bla*_SHV-98_ *bla*_SHV-145_ *bla*_SHV-179_ *bla*_SHV-194_ *bla*_SHV-199_ *bla*_CTX-M-15_	*bla*_SHV-11_ *bla*_SHV-67_	*bla*_SHV-28_ *bla*_SHV-106_
Fosfomycin	*fosA5*	*fosA6*	*fosA5*	*fosA5*	*fosA5*	*fosA6*
Quinolones	OqxA OqxB	OqxA OqxB	OqxA OqxB	OqxA OqxB	OqxA OqxB	OqxA OqxB
Virulence genes	*fyuA*	*ompA*	*ompA*	*fyuA*	*ompA*	*ompA*
	*ybtE*	*entA*	*entA*	*ybtE*	*entA*	*entA*
	*ybtT*	*entB*	*entB*	*ybtT*	*entB*	*entB*
	*ybtU*	*fepC*	*fepC*	*ybtU*	*fepG*	*fepC*
	*irp1*	*yagV/ecpE*	*yagV/ecpE*	*irp1*	*fepC*	*yagV/ecpE*
	*irp2*	*yagW/ecpD*	*yagW/ecpD*	*irp2*	*yagV/ecpE*	*yagW/ecpD*
	*ybtA*	*yagX/ecpC*	*yagX/ecpC*	*ybtA*	*yagW/ecpD*	*yagX/ecpC*
	*ybtP*	*yagY/ecpB*	*yagY/ecpB*	*ybtP*	*yagX/ecpC*	*yagY/ecpB*
	*ybtQ*	*yagZ/ecpA*	*yagZ/ecpA*	*ybtQ*	*yagY/ecpB*	*yagZ/ecpA*
	*ybtX*		*ykgK/ecpR*	*ybtX*	*yagZ/ecpA*	*ykgK/ecpR*
	*ybtS*			*ybtS*	*ykgK/ecpR*	
	*ompA*			*ompA*		
	*entA*			*entA*		
	*entB*			*entB*		
	*fepC*			*fepC*		
	*yagV/ecpE*			*yagV/ecpE*		
	*yagW/ecpD*			*yagW/ecpD*		
	*yagX/ecpC*			*yagX/ecpC*		
	*yagY/ecpB*			*yagY/ecpB*		
	*yagZ/ecpA*			*yagZ/ecpA*		
	*ykgK/ecpR*			*ykgK/ecpR*		
ST	16	340	1,269	16	147	15

### Plasmid-mediated resistance genes

The CRKP isolates harbored between two to four plasmids, with sizes ranging from 188,688 to 6,141 base pairs (Table 4). Plasmids were designated by placing the letter p in front of the isolate name followed by an underscore and a numerical value (eg pKPH1_1). The replicon type of a 82,102 bp plasmid found in KPA2 was unidentified. The presence of numerous replicon types within a plasmid was observed in KPH1 and KPA1, which contained IncFIA, IncFIB, and IncFII; KPH2 contained IncFIA(HI1) and IncR; and KPA3 contained IncFIB(pQil), IncFII(K) and IncHI1B. In the human isolates, plasmids pKPH1_1, pKPH3_1, and ColKP3 plasmid pKPH4_3 were confirmed to contain *bla*_OXA-1_, *bla*_OXA-9_, and *bla*_OXA-232_, respectively. The gene *bla*_NDM-1_ encoding NDM was identified on plasmids pKPH1_2, pKPH3_2, and pKPA3_1 in two isolates from humans and one from a dog, respectively. The *bla*_OXA-232_ gene was identified on the plasmid pKPA2_4 and on the ColKP3 plasmid pKPA1_3, in the two respective dog isolates; ColKP3 also was found in human KPH4 and had the same size. Notably, *bla*_OXA-48_ was identified only in plasmid pKPA1_2. All the isolates contained gentamicin-resistance genes, and five of the six contained quinolone-resistance genes. Moreover, the *mcr 3.5* gene was identified only on the IncFII plasmid in human pKPH4_1, while the IncFII plasmid in the dog isolates only contained *bla*_OXA-48_.

**Table 4:** Comparison of antimicrobial resistance genes and plasmid replicon types from *K. pneumoniae* of human and dog origin.

	Human isolates and their plasmids							Dog isolates and their plasmids								
	KPH1		KPH3		KPH4			KPA1			KPA2				KPA3	
	pKPH1_1 (132,031 bp)	pKPH1_2 (125,297 bp)	pKPH3_1 (108,736 bp)	pKPH3_2 (41,190 bp)	pKPH4_1 (77,911 bp)	pKPH4_2 (42,965 bp)	pKPH4_3 (6,141 bp)	pKPA1_1 (111,444 bp)	pKPA1_2 (68,908 bp)	pKPA1_3 (6,141 bp)	pKPA2_1 (188,688 bp)	pKPA2_2 (160,519 bp)	pKPA2_3 (82,102 bp)	pKPA2_4 (6,141 bp)	pKPA3_1 (175,819 bp)	pKPA3_2 (121,101 bp)
Accession No. (*NCBI)	CP102547	CP102548	CP102553	CP102555	JAOAOE010000005	JAOAOE010000006	JAOAOE010000009	CP101878	CP101880	CP101881	CP102988	CP102989	CP102990	CP102991	CP102994	CP102995
Plasmid replicon types	IncFIB(pQil) IncFII(K)	IncFIA IncFIB IncFII	IncFIA(HI1) IncR	IncN2	IncFII	IncN	ColKP3	IncFIA IncFIB IncFII	IncFII	ColKP3	IncFIB(K)	IncC	Unknown	ColKP3	IncFIB(pQil) IncFII(K)	IncFIB(K) IncHI1B
Resistance genes																
β-lactam	*bla*_CTX-M-15_ *bla*_TEM-1A_ *bla*_OXA-9_	*bla*_NDM-1_	*bla*_CTX-M-15_ *bla*_TEM-1B_ *bla*_OXA-1_ *bla*_SHV-12_	*bla*_NDM-1_		*bla*_TEM-1B_	*bla*_OXA-232_		*bla*_OXA-48_	*bla*_OXA-232_	*bla*_DHA-1_	*bla*_CMY-2_	*bla*_TEM-1B_ *bla*_CTX-M-3_	*bla*_OXA-232_	*bla*_NDM-1_	
Aminoglycoside	*aadA1* *aac(6′)-Ib*	*aadA2*	*aph(3″)-Ib* *aac(3)-IIa* *aph(6)-Id*		*aac(3)-*VIa *aadA1*			*aadA2*			*aph(3′)-Ia*	*aac(6′)-Ib3* *aph(6)-Id* *aph(3″)-Ib* *ant(2″)-Ia* *aac(6′)-Ip*	*aadA16* *rmtB*		*rmtF*	
Quinolones	*qnrB1* *aac(6′)-Ib-cr*		*aac(6′)-Ib-cr*		*qnrS1*	*qepA1* *qepA2* *qepA4*					*qnrB4*	*qnrVC4* *aac(6′)-Ib-cr*	*qnrS1*		*qnrB1* *aac(6′)-Ib-cr*	
Trimethoprim		*dfrA12*	*dfrA14*					*dfrA12*					*dfrA27*			
Sulphonamide		*sul1*	*sul2*					*sul1*				*sul2*	*sul1*			
Tetracycline		*tet(B)*	*tet(A)* *tet(D)*					*tet(B)*				*tet(A)* *tet(M)*				*tet(D)*
Phenicol			*catB3* *catA2*									*floR* *cmlA1*				*catA1*
Colistin					*mcr-*3.5											
Macrolide											*mph(A)*					
Rifampicin													*ARR-3*		*ARR-2*	
Biocide		*qacE*						*qacE*			*qacE*	*qacL*	*qacE*			

^*^National Center for Biotechnology Information database.

### Genomic comparison and phylogenetic SNP tree analysis

**Figure 1:**
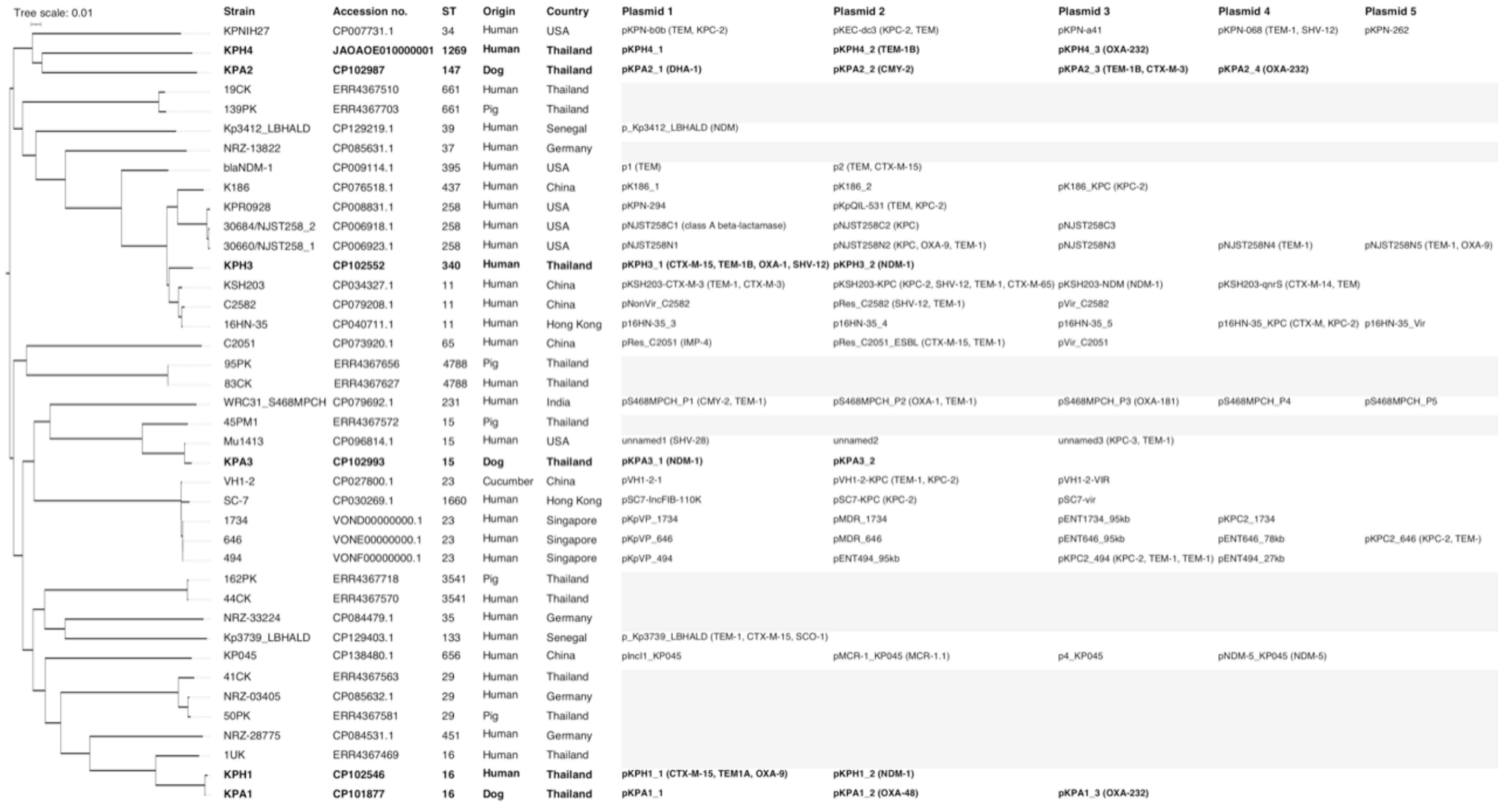
Phylogenetic single nucleotide polymorphisms (SNP) tree of 40 isolates of *Klebsiella pneumoniae* producing carbapenemase (KPC), including the six isolates from the current study (marked in bold), and 10 previous isolates from Thailand. Where available, the sequence type (ST), accession number, species and country of origin of the isolates, plasmid names, numbers, and carbapenem-resistance genes are presented. The gray highlight represents not identified or unavailable.

**Figure 2:**
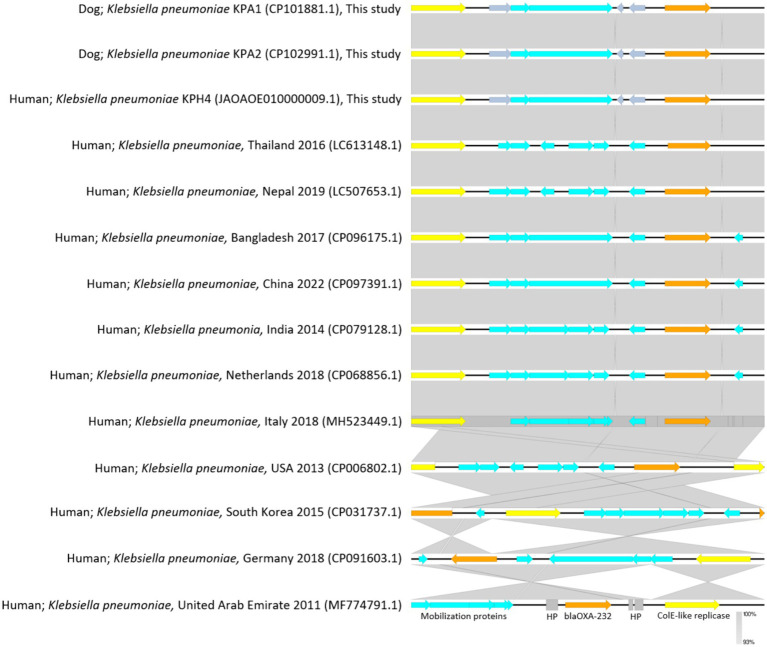
Plasmidome comparative analysis of sequenced regions of ColKP3 containing *bla*_OXA-232_ in *Klebsiella pneumoniae* from accession No. CP101881.1; dog Thailand 2020 (KPA1), No. CP102991.1; dog Thailand 2020 (KPA2), and No. JAOAOE010000009.1; human Thailand 2020 (KPH4) compared with *K. pneumoniae* from humans in each of the countries for *bla*_OXA-232_ (size; 6,141 bp) from No. LC613148.1; human Thailand 2016, No. LC507653.1; human Nepal 2019, No. CP096175.1; human Bangladesh 2017, No. CP097391.1; human China 2022, No. CP079128.1; human India 2014, No. CP068856.1; human the Netherlands 2018, No. MH523449.1; human Italy 2018, No. CP006802.1; human USA 2013, No. CP031737.1; human South Korea 2015, No. CP091603.1; human Germany 2018, and No. MF774791.1; human United Arab Emirates 2011. The gray area indicates the blast identities, and the percentage of identity is indicated in the legend. Open arrows represent coding sequences (blue for mobilization proteins, orange for *bla*_OXA232_, yellow for ColK-like replicase). The arrow size is proportional to the gene length. The image was generated using EasyFig with default parameters.

For genomic comparisons and phylogenetic tree analysis the genome sequences of the three dog isolates and three human isolates were aligned with those of 34 *K. pneumoniae* carbapenemase containing (KPC) strains from Thailand and other countries. These showed different plasmids in relation to their ST, source, and country of origin (Figure 1). The Thai ColKP3 plasmids were very similar to each other, and they showed approximately 93 percent similarity to the nucleotide sequences of the plasmids from human strains possessing *bla*_OXA-232_ (Figure 2).

**Figure 3:**
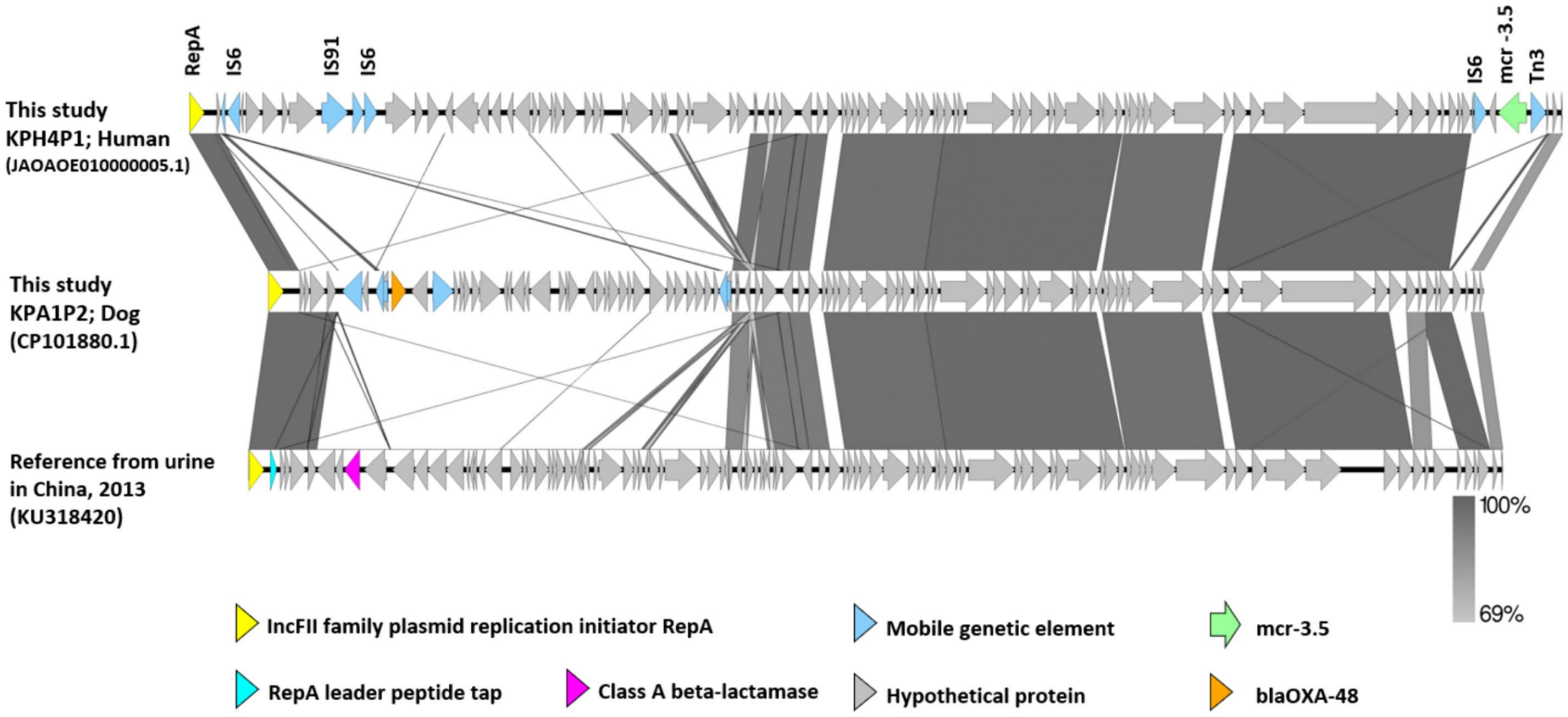
The genetic environment of *mcr-3.5* and *bla*_OXA-48_ genes from carbapenem resistant *K. pneumoniae* isolates KPH4 and KPA1 containing an IncFII plasmid from this study compared with a reference plasmid. The gray area indicates the blast identities, and the percentage of identity is indicated in the legend. Open arrows represent coding sequences (green for *mcr-3.5*, orange for *bla*_OXA48_, purple for class A beta-lactamase and blue for mobile genetic elements). The arrow size is proportional to the gene length. The image was generated using EasyFig with default parameters. Accession no. JAOAOE010000005.1; human Thailand 2020 (KPH4P1) and accession no. CP101881.1; dog Thailand 2020 (KPA1P2) compared with a reference strain from a urine sample (IncFII; accession No. KU318420 from China 2013).

A comparison of the genetic environment of the *mcr-3.5* cassette and *bla*_OXA-48_ from IncFII plasmids pKPH4_1 and pKPA1_2 in isolates pKPH4 and pKPA1 respectively, and the reference plasmid from a strain from a human urine sample (accession No. KU318420 from China 2013) is presented in Figure 3. The *mcr-3.5* and *bla*_OXA-48_ genes were not in the core structure of the plasmid and were flanked by insertion sequence 6 (IS6), transposon 3 (Tn3) and other mobile genetic elements (Figure 3). The reference plasmid lacked these resistance genes.

### Conjugation of plasmids

The six transconjugants had conjugation rates ranging from 8 × 10^−3^ to 1.7 × 10^−7^ and were both genetically and phenotypically resistant to sodium azide and meropenem. KPA1 had the highest conjugation rate (Table 5).

**Table 5:** Conjugation efficacy of *E. coli* J53 acquiring genes for carbapenem resistance from *Klebsiella pneumoniae.*

Donor (*Klebsiella pneumoniae*)	Source	Recipient *E. coli* J53 (Transconjugants)			
		MIC (μg/mL)			Conjugation efficiency
		Imipenem	Meropenem	Ertapenem	
KPH1	Human	>16	>16	>2	8 × 10^−7^
KPH3	Human	>16	>16	>2	4.13 × 10^−6^
KPH4	Human	0.25	0.5	>2	4.2 × 10^−6^
KPA1	Dog	>16	>16	>2	1.7 × 10^−3^
KPA2	Dog	2	1	>2	4.5 × 10^−6^
KPA3	Dog	>16	>16	>2	4.3 × 10^−6^

## Discussion

The emergence and spread of carbapenem-resistant *Klebsiella pneumoniae* strains are of significant concern due to their limited treatment options and potential for causing severe infections (1). In this study, we characterized six CRKP isolates from humans and dogs with UTIs, focusing on their antimicrobial susceptibility, genomic features, and similarity of their plasmidome, focusing on plasmid-mediated resistance genes. The SNP tree indicated that KPH1 and KPA1 shared ST16 and were indistinguishable apart from their plasmid content (Figure 1). This suggests that these closely related isolates may be transmissible between humans and dogs. The other four isolates were dispersed across the SNP tree and were related to isolates from various sources and countries.

Antimicrobial susceptibility testing confirmed that all six isolates were phenotypically and genotypically resistant to ertapenem, with four showing additional resistance to imipenem and meropenem. The differential resistance pattern of *K. pneumoniae* to ertapenem compared to imipenem and meropenem can be attributed to several factors. Although all three antibiotics belong to the carbapenem class (39), there are subtle differences in their chemical structures and mechanisms of action, which can affect their effectiveness against resistant bacteria. *K. pneumoniae* can acquire various resistance mechanisms, including the production of carbapenemases that degrade or modify carbapenem antibiotics. Different carbapenemases may have varying affinities for different carbapenem drugs, leading to differences in resistance profiles (40). Importantly, none of the isolates exhibited tigecycline resistance, highlighting its potential as an alternative treatment option for CRKP infections (41).

Genomic characterization of the CRKP isolates revealed the presence of different carbapenemase genes, including NDM-1 and OXA variants. The presence of these genes indicates the potential for high-level carbapenem resistance in *K. pneumoniae* isolates and highlights the presence of specific resistance mechanisms that contribute to the spread and persistence of carbapenem resistance in isolates from animal and human patients (22).

The isolates shared common replicon types, such as InFIA and IncFIB, indicating the potential for plasmid-mediated horizontal gene transfer among them. The InFIA replicon type is associated with the plasmid family IncF, which is known for its broad host range and ability to transfer between different bacterial species. IncF plasmids often carry multiple resistance genes and are frequently found in clinical isolates of *K. pneumoniae* (27, 42, 43). In particular, InFIA and IFB plasmids have been associated with the dissemination of carbapenem resistance genes, such as *bla*_NDM-1_, in *K. pneumoniae* strains (22). Unfortunately, their identification in *K. pneumoniae* is a common finding, especially among multidrug-resistant strains and those carrying carbapenemase genes. Their presence in our isolates suggests the potential for horizontal gene transfer and the dissemination of resistance genes among bacterial populations in Thailand.

Notably, a small ColKP3 plasmid was found in both human and dog isolates, suggesting a potential reservoir for the dissemination of carbapenem resistance genes between humans and animals. The ColKP3 replicon plasmid has been implicated in the dissemination of carbapenemase encoding genes such as *bla*_OXA-232_ (44) that can hydrolyze carbapenems, leading to resistance. The presence of the ColKP3 replicon plasmid carrying carbapenemase genes could contribute to the spread of carbapenem resistance among *K. pneumoniae* strains. The ColKP3 replicon plasmid belongs to the group of small plasmids known as ColE-like plasmids (10) that are often self-transmissible and can replicate autonomously. Their small size facilitates their transfer between bacteria, allowing for the rapid dissemination of resistance genes (45).

Analysis of the chromosomes of the six isolates showed that they had similar characteristics, including comparable CG content, number of CDSs, rRNAs, and tRNAs. The presence of *bla*_SHV_ genes encoding β-lactam resistance and *fosA* genes conferring fosfomycin resistance was observed on the chromosomes of all isolates. Additionally, the combination of *oqxA* and *oqxB* genes responsible for quinolone resistance was identified. This finding suggests that these resistance determinants are part of the inherent genetic makeup of the bacteria. In this case, their presence on the chromosome may not necessarily indicate the immediate potential for horizontal gene transfer. In clinical isolates the *oqxA and oqxB* genes generally locate on the chromosome and/or plasmids flanked by IS26-like elements, conferring low to intermediate resistance to quinoxalines, quinolones, tigecycline, and nitrofurantoin. These have the potential to co-spread with other antimicrobial resistance genes (*bla*_CTX-M_, *rmtB* and *aac(6′)-Ib* etc.) (46). The *bla*_SHV_ genes encode beta-lactamase enzymes that can hydrolyze and inactivate beta-lactam antibiotics, including penicillins and some cephalosporins. The presence of *bla*_SHV_ genes on the chromosome of *K. pneumoniae* strains may indicate the intrinsic resistance of these bacteria to beta-lactam antibiotics (47). However, the presence of plasmid-borne non-ESBL-encoding *bla*_SHV_ is a risk factor for the development of an ESBL-positive phenotype, despite the same gene being present on the chromosome (4). This can impact the choice of antibiotics for treatment, as these strains may not respond well to certain drugs.

The ST16 clonal type of *K. pneumoniae* has proliferated globally and is commonly associated with multidrug-resistant strains carrying enzymes that produce NDM-1, CTX-M-15, and OXA-232 that cause resistance to crucial medicines (48). The spread of these antibiotic-resistant bacteria, particularly between animals and humans, poses a serious threat to public health since it reduces treatment options and can result in treatment failures (1). The two ST16 isolates from a human and a dog in this study had highly similar profiles of resistance and virulence genes on their chromosomes, including genes encoding ESBL-generating enzymes. On the other hand, *bla*_NDM-1_ and *bla*_OXA-9_ genes were identified on plasmids in the human isolates, whereas *bla*_OXA-48_ genes were found in the dog isolates. The identification of these specific resistance genes on plasmids in human and dog isolates suggests the involvement of mobile genetic elements in gene transmission. This finding emphasizes the potential for genetic exchange and the spread of resistance genes via mobile genetic components, but not chromosomal reliance (49).

The plasmid analysis revealed the presence of two to four plasmids in each CRKP isolate, with varying sizes and replicon types. Interestingly, several plasmids showed the coexistence of multiple replicon types, indicating the potential for diverse plasmid compatibility and dissemination mechanisms. The plasmids harbored various resistance genes, including *bla*_OXA-1_, *bla*_OXA-9_, *bla*_OXA-48_, *bla*_OXA-232_, and *bla*_NDM-1_. Prior to this study, examination of the genomic characteristics of multidrug-resistant ST11 *K. pneumoniae* isolates and their plasmids demonstrated the existence of several plasmids of various sizes and replicon types, indicating variable plasmid compatibility and dissemination mechanisms (50). In a previous study in Thailand, 12 of 15 *K. pneumoniae* ST16 isolates with chromosomal clonality contained both *bla*_NDM-1_ and *bla*_OXA-232_, indicating that these plasmids were widely distributed in *K. pneumoniae* (16). In comparison, all but one of 13 ST231 isolates in that study only harbored *bla*_OXA-232_ (16). Another study in clinically affected humans from Thailand also described *K. pneumoniae* ST16 as a clone that had virulence genes that were similar to those of other highly transmissible *K. pneumoniae* that carried *bla*_NDM-1_ and *bla*_OXA-232_ (51). When considering ST15, dog strain KPA3 in the current study showed resistance associated with plasmid pKPA3_1 (NDM-1) and pKPA3_2 (Figure 1). In another study in Thailand, pig strain 45 PM1 showed no plasmid-associated resistance determinants, whereas human strain Mu1413 isolated in the USA carried resistance determinants on plasmids unnamed1 (SHV-28), unnamed2, and unnamed3 (KPC-3, TEM-1) (52). For ST16, in the current study dog strain KPA1 carried resistance determinants on plasmids pKPA1_1, pKPA1_2 (OXA-48), and pKPA1_3 (OXA-232). Moreover, the closely related human strain KPH1 carried resistance determinants on plasmids pKPH1_1 (CTX-M-15, TEM1, OXA-9) and pKPH1_2 (NDM-1). In the earlier study in Thailand, human strain 1UK exhibited no resistance associated with the plasmids identified in other Thai isolates (52). These findings highlight the genetic relatedness and varied distribution of AMR genes among *K. pneumoniae* isolates, emphasizing the interconnectedness of human, animal, and environmental sources and transmission of resistance mechanisms across diverse hosts and environments.

Notably, the *mcr-3.5* gene conferring colistin resistance was identified on an IncFII plasmid in one of the human isolates (KPH4), indicating the coexistence of multiple resistance mechanisms. The *mcr*-*3.5* gene is a variant of the mobile colistin resistance gene (*mcr*) family (53). It encodes resistance to colistin, an antibiotic belonging to the polymyxin class. The *mcr*-3.5 gene was first identified in an *Escherichia coli* strain isolated from pig feces in China (54). It has been reported to be associated with IncFII plasmids, which are known to have a broad host range and can be transferred between different bacterial species (53). The presence of colistin resistance genes on IncFII plasmids supports the notion of multiple resistance mechanisms coexisting in *K. pneumoniae* strains. The findings will help guide the development of effective surveillance strategies and infection control measures, ultimately contributing to the preservation of public health and the improvement of patient outcomes.

In conclusion, this study offers valuable insights into the antimicrobial susceptibility, genomic characteristics, and plasmid-mediated resistance genes of CRKP isolates from unrelated humans and dogs with UTIs in Thailand. However, the limited number of isolates investigated in this study and the restricted clinical data rendered it difficult to determine whether the outbreak inside the animal and human hospital developed and evolved over time. Although human isolate KPH1 and dog isolate KPA1 both belonged to ST16 and were indistinguishable on the SNP tree, they had different plasmid types and had no known direct epidemiological connections. Nevertheless, the demonstration that human and dog isolates shared antimicrobial resistance and virulence genes on their chromosomes and had genes encoding NDM and OXA enzymes on plasmids that are identical to ColKP3 indicates the potential for interspecies transmission, and emphasizes the need for surveillance and control measures to prevent the spread of CRKP strains. Moreover, when the six isolates in the current study where compared to global isolates of *K. pneumonia*, these showed a variety of divergent genomes and plasmid types. There is a need for additional research into the precise mechanisms underlying the transmissibility and pathogenicity of CRKP strains and their potential effects on human and animal health.

## Data availability statement

The datasets presented in this study can be found in online repositories. The names of the repository/repositories and accession number(s) can be found in the article/Supplementary material.

## Ethics statement

Ethical approval was not required for the study involving animals in accordance with the local legislation and institutional requirements because all the isolates we used were obtained from the frozen stock of the Pathogen Bank in our institute. These were clinical isolates that were submitted for diagnostic purposes as a routine procedure. Written informed consent was obtained from the owners for the participation of their animals in this study.

## Author contributions

RP: Writing – review & editing, Writing – original draft, Validation, Software, Methodology, Investigation, Formal analysis, Data curation. NK: Writing – original draft, Resources, Methodology, Investigation, Formal analysis, Data curation. NPh: Writing – review & editing, Visualization, Methodology, Investigation, Formal analysis, Data curation. TW: Writing – review & editing, Validation, Software, Resources, Methodology. WN: Writing – review & editing, Resources, Methodology, Data curation. TC: Writing – review & editing, Resources, Methodology, Data curation. DH: Writing – review & editing, Supervision, Conceptualization. NPr: Writing – review & editing, Writing – original draft, Validation, Supervision, Resources, Project administration, Methodology, Investigation, Funding acquisition, Data curation, Conceptualization.

## Footnotes

^1^ https://usegalaxy.eu

^2^ https://cge.cbs.dtu.dk

^3^ https://itol.embl.de
