# Supplementary material for: Genomic characterization of carbapenem and colistin-resistant Klebsiella pneumoniae isolates from humans and dogs
Source: Front Vet Sci. 2024 May 21;11:1386496. doi: 10.3389/fvets.2024.1386496 (PMC11148352; doi:10.3389/fvets.2024.1386496)
Supplement: Supplementary file 1 [file Image_1.pdf]

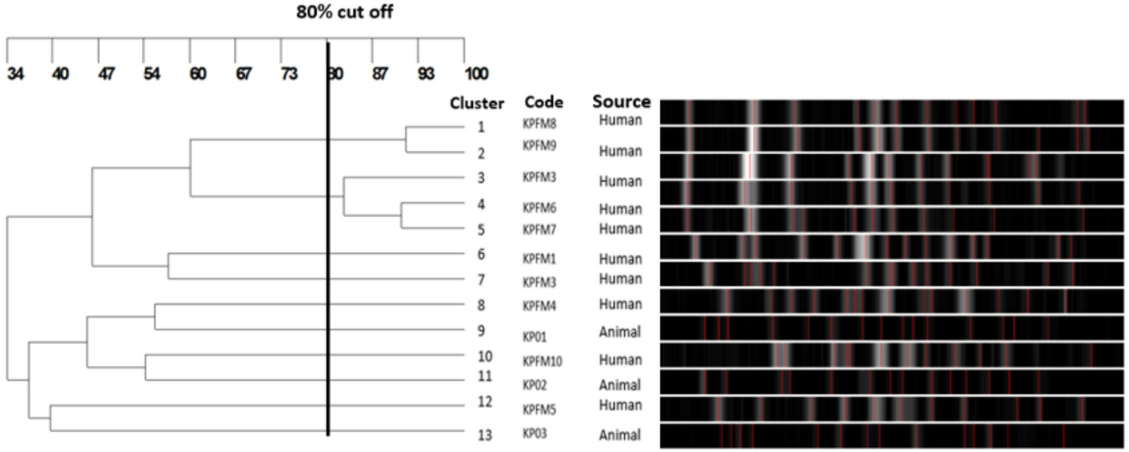


Supplementary Figure. Pulsed-field gel electrophoresis (PFGE) of Carbapenem resistant *Klebsiella pneumonieae* from canine and human isolates.
